# Cerebellar venous thrombosis mimicking a cerebellar tumor due to polycythemia vera: a case report

**DOI:** 10.1186/s12883-021-02261-1

**Published:** 2021-06-16

**Authors:** Hongfeng Wen, Di Jin, Yu Chen, Bin Cui, Tianyi Xiao

**Affiliations:** 1 Department of Neurology, Aerospace Center Hospital, Beijing, China; 2 Department of Radiology, Aerospace Center Hospital, Beijing, China

**Keywords:** Cerebral venous thrombosis, Polycythemia vera, Cerebellum, Tumor, Venous infarction

## Abstract

**Background:**

Cerebral venous thrombosis (CVT) occurs rarely in the general population and is frequently associated with confused clinical findings and delayed diagnosis. Isolated cerebellar cortical vein thrombosis is a very rare phenomenon.

**Case presentation:**

This report describes a case with CVT, which is manifested as space-occupying lesions of the cerebellar hemisphere and mimics a cerebellar tumor at the beginning. The diagnosis of CVT was finalized given the laboratory and brain biopsy findings. The etiology may be related to polycythemia vera with Janus Kinase 2 V617F mutation.

**Conclusion:**

Isolated cerebellar vein thrombosis should be considered when swelling and enhancing cerebellar lesions are detected. Polycythemia vera, especially with a positive JAK2 V617F mutation, may be a rare risk factor for CVT.

## Background

Cerebral venous thrombosis (CVT) is a cerebrovascular disorder. It is associated with the occlusion of dural sinuses and cerebral veins and results from the blocked cerebral venous reflux, thus usually causing non-hemorrhagic and hemorrhagic stroke and being a serious, even potentially life-threatening, disease. [1, 2] CVT is a rare condition and represents approximately 1–4% of all intracranial vein thrombosis [1, 2]. The common risk factors for CVT include inherited thrombophilia (e.g., factor V Leiden mutation, protein C and S deficiency), acquired prothrombotic state (e.g., pregnancy, puerperium and postoperative period), systemic disease (e.g., Behcet’s syndrome and systemic lupus erythematosus), neoplasia, oral contraceptives and local causes [3]. Polycythemia vera (PV), a rare form of blood cancer, rarely causes cerebellar cortical vein thrombosis. And a previous study suggest that CVT is poorly associated with myeloproliferative neoplasms (especially the polycythemia vera, PV) [4]. This report describes a case of cerebellar cortical vein thrombosis due to PV in a patient. The patient had a history of PV with Janus Kinase 2(JAK2) V617F mutation and was initially suspected to have a cerebellar tumor. CVT was finally diagnosed according to laboratory and brain biopsy findings.

## Case presentation

**Fig. 1:**
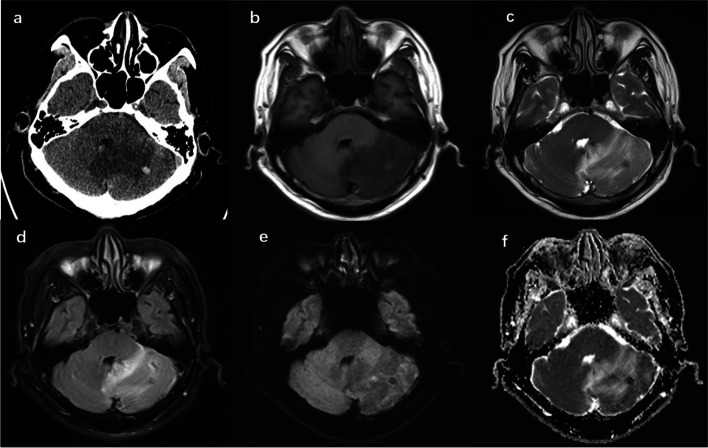
**a** CT imaging showing a lesion in the Left cerebellar hemisphere, with a large low-density area in the left cerebellar hemisphere and vermian and a small, round-like, and high-density area in the left cerebellar hemisphere; **b** MRI imaging showing hypointense on T1WI; **c** MRI imaging showing hyperintense on T2WI; **d** MRI imaging showing FLAIR; **e** MRI imaging showing slightly hypointense signal on DWI; and f MRI imaging showing hyperintense on ADC

**Fig. 2:**
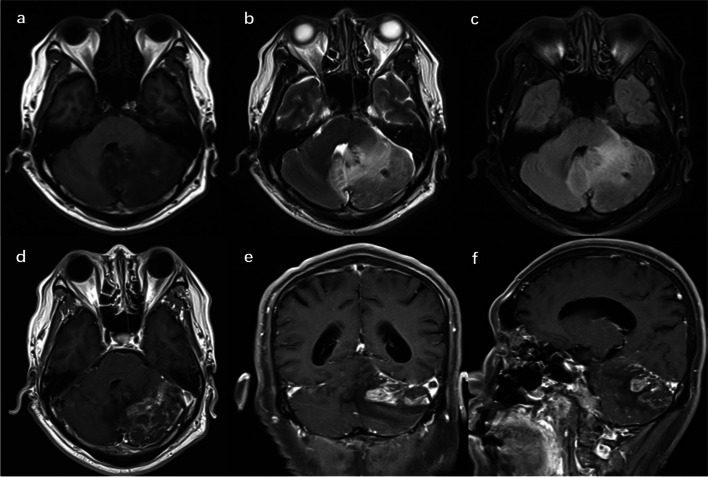
**a**-**c** MRI imaging showing an aggravation of the lesion. **a** Hyperintense on T1WI; **b** Hyperintense on T2WI; and **c** FLAIR; **d**-**f** Contrast-enhanced imaging showing heterogeneous enhancement of the lesion. **d** Axial T1WI with gadolinium; **e** Coronal T1WI with gadolinium and **f** Sagittal T1WI with gadolinium

A 62-year-old male patient was presented with recurrent vertigo, vomiting, ataxia and mild dysarthria for 14 h. The patient had a history of hypertension and hyperuricemia with regular medication control. The patient was diagnosed with PV by bone marrow biopsy via PCR detection four years prior to presentation and received long-term oral hydroxyurea therapy. Neurological examination suggested that the patient was lethargic, without cranial nerve palsy or meningeal irritation. Laboratory results showed that hemoglobin was 183 g/L (normal range, 130–170 g/L, grams per liter), hematocrit was 0.678 (normal range, 0.4–0.5), red cell count was 7.31 × 10^12^/L (normal range, 4.3–5.8 × 10^12^/L), white cell count was 15.56 × 10^9^/L (normal range, 3.5–5.5 × 10^9^/L) and 256 × 10^9^/L platelets (normal range, 125–350 × 10^9^/L). The results of biochemical and clotting tests were normal. Computerized tomography (CT) showed a large low-density area in the left cerebellar hemisphere and vermian. A small, round-like, and high-density area was observed in the left cerebellar hemisphere (Fig. 1a). Magnetic resonance imaging (MRI) revealed the hypointense on T1-weighted images (T1WI), hyperintense on T2-weighted images (T2WI), fluid-attenuated inversion recovery (FLAIR), slightly hypointense signal on diffusion-weighted imaging (DWI) and hyperintense on apparent diffusion coefficient (ADC) (Fig. 1b-f). Compressed pons and medulla oblongata were found. MRI showed an increased extent of lesion four days later, accompanied by obvious mass effect (Fig. 2a-c). On contrast enhanced imaging, lesions showed heterogeneous enhancement, with many dilated cortical veins (Fig. 2d-f).

**Fig. 3:**
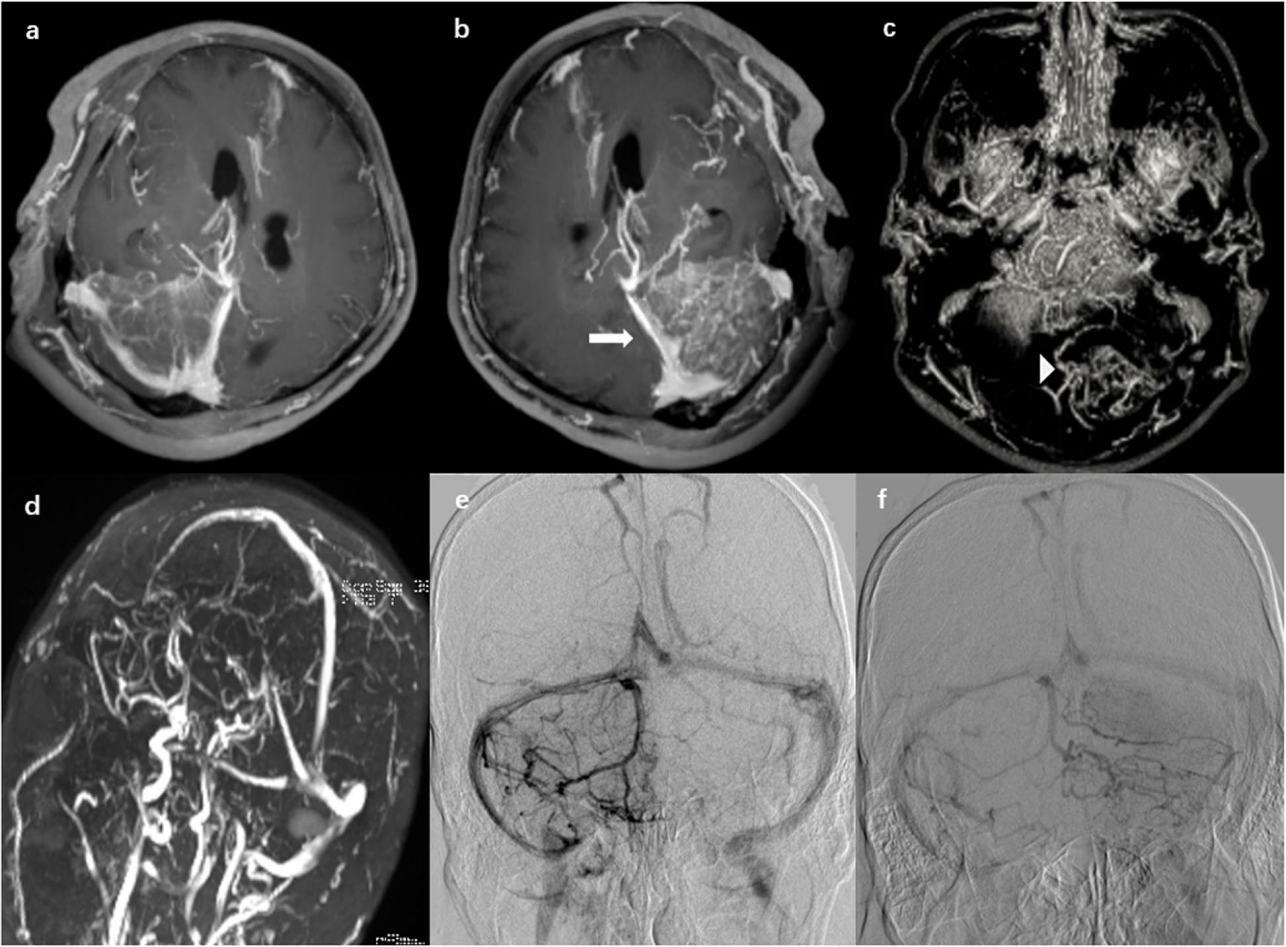
**a** Relatively normal anatomical venous structure of the right cerebellum releaved by 3D T1-MPRAGE; **b** The left tentorium of cerebellum displayed by 3D T1-MPRAGE, with more evidently disorganized vessels (arrow) than those in the other side; **c** Abnormally increased and disorganized venous vessels in the inferior portion of the left cerebellar hemisphere (arrowhead); **d** TOF MRV imaging showing no characteristic changes, with no filling defects in the dural venous sinuses or in major vessels of the deep cerebral venous system; **e **The tortuous and dilated veins in the left cerebellar hemisphere draining through the right transtentorial sinus and showing delayed drainage; **f** The unvisualized left transtentorial sinus

Since the obvious mass effect of the lesion, a neoplastic disease was the initial diagnostic hypothesis, therefore the patient underwent a robot-assisted stereotactic brain biopsy under general anesthesia. Pathological examination demonstrated the evidence of hemorrhagic infarction, while no neoplastic cells or vasculitis were found. Lumbar puncture showed that the cerebrospinal fluid pressure was 165 mmH_2_O, with an increased protein content (about 106.80 mg/dL, normal range, 15–45 mg/dL). No significant changes in glucose, chloride, or cell counts were detected. For further diagnose, a contrast-enhanced three-dimensional T1 magnetization-prepared rapid acquisition gradient-echo (3D T1-MPRAGE) imaging was conducted in the patient. The obtained images revealed more evidently disorganized vessels in the left tentorium of cerebellum than those in the other side. Besides, abnormally increased and disorganized venous vessels in the inferior portion of the left cerebellar hemisphere were observed (Fig. 3a-e). These findings suggested the possibility of venous infarction.

Considering the venous infarction, the patient received anticoagulant therapy with oral dabigatran etexilate (110 mg twice daily) at 2 weeks after biopsy. After a 24-day anticoagulant therapy, enhanced MRI showed the enhancement of the lesion and the obviously decreased mass effect. Data from THE time-of-flight (TOF) magnetic resonance venography (MRV) showed no filling defects in the dural venous sinuses or in major vessels of the deep cerebral venous system. Meanwhile, the symptoms of vertigo and ataxia were obviously relieved. After a 54-day anticoagulant therapy, digital subtraction angiography (DSA) showed the normal arteries supply to the posterior fossa without arteriovenous malformations. The left transtentorial sinus was not visualized, while the veins in the left cerebellar hemisphere were tortuous and dilated, which finally drained through the right transtentorial sinus with delayed drainage (Fig. 3f-i). Taken together, the results of DSA and the improvement of clinical symptoms suggested the cerebellar venous sinus thrombosis.

**Fig. 4:**
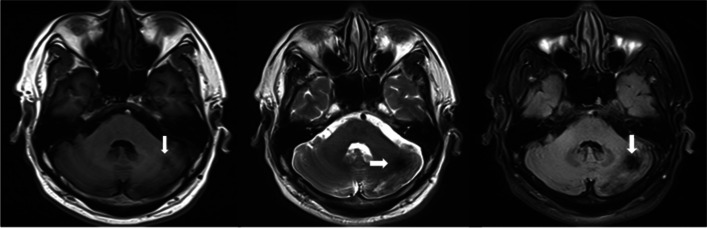
MRI scan showing the resolution of the cerebellar lesion except the traces after biopsy (arrow) after a 4-month anticoagulation therapy

Bone marrow biopsy revealed that the patient was positive for JAK2 V617F mutation. We, therefore, speculated PV as a predisposing factor for the occurrence of thrombosis. The follow-up MRI scan after a 4-month anticoagulation therapy demonstrated the resolution of cerebellar lesion except the traces after biopsy (Fig. 4a-c).

## Discussion and Conclusions

CVT is a rare but serious condition, with an annual incidence of 3–4 cases per million [5]. A wide range of clinical presentations occurs in patients with this condition. The most common symptom is severe headache, which present in more than 90% of cases [6]. Focal neurological deficit (46%), papilledema (41%), seizures (47%) and decreased consciousness (39%) are also the common features [7]. The patient in the current report was presented with vertigo, recurrent vomiting, ataxia and mild dysarthria, possibly related to the lesions of cerebellum. The initial MRI scan showed the malignant vasogenic edema with minor parenchymal hemorrhage and the mass-like enhancement, which mislead us to the probable diagnosis of neoplastic disease at the beginning. Subsequent brain biopsy ruled out this possibility. Our findings are consistent with the previous study reporting that CVT can indeed be misdiagnosed as a cerebellar tumor due to its uncommon frequency ratios in subtentorium [8]. The massive vasogenic edema could be attributed to the occurrence of PV. PV can increase the blood viscosity, which causes the development of thrombosis. Thrombosis of the cerebral veins and sinuses then increases pressure in veins and capillaries, leading to a decreased cerebral perfusion pressure, ischemic injury and cytotoxic edema. Besides, disruption of the blood–brain barrier leads to vasogenic edema. [5]

A wide range of etiologies are involved in the occurrence and development of CVT. Predisposing factors include inherited thrombophilia, acquired prothrombotic state (including pregnancy and the puerperium), blood dyscrasias (e.g., thrombocythemia), drugs (e.g., contraceptives) and local factors (e.g., head injury, arteriovenous malformation, meningitis, middle ear infections and facial infections) [7]. Myeloproliferative neoplasms (including PV, essential thrombocythemia, and primary myelofibrosis) are possibly associated with the occurrence of CVT, with a prevalence of 3–7% in individuals with CVT [9]. Despite the rare diagnosis of CVT, patients are more likely to concomitant with PV than the other two subtypes [4]. PV features clonal proliferation of hematopoietic stem cells which leads to abnormal increasement and accumulation of circulating red blood cells, white blood cells and platelets within the circulation, thus increasing blood viscosity and decreasing blood flow velocity [10]. PV, therefore, causes stasis of blood that results in hyperviscosity and eventually the development of thrombosis. Thrombosis of the cerebral veins and sinuses increases venular and capillary pressure, which decreases the cerebral perfusion pressure and causes ischemic injury and cytotoxic edema. Venous and capillary rupture culminates in parenchymal hemorrhage. The disruption of the blood–brain barrier, associated with cytotoxic edema, leads to vasogenic edema. Thrombosis of cerebral sinuses impairs cerebrospinal fluid (CSF) absorption, thus increasing the intracranial pressure. The increased intracranial pressure further aggravates venular and capillary hypertension and contributes to parenchymal hemorrhage, vasogenic edema and cytotoxic edema [5]. Aging and high hematocrit increase the risk of arterial and venous vascular events especially in the cerebral circulation, with the frequency of venous events far less than that of arterial ones [11, 12]. Besides, our patient is positive for JAK2 V617F mutation, which is considered as an independent risk factor for thrombosis [13]. Although the lower appearance of JAK2 V617F mutation than splanchnic vein thrombosis (mean prevalence of 32.7%), its global prevalence is unnegligible (3.9%), which suggesting the value of mutation screening in patients with and without overt myeloproliferative neoplasms. [9]

CVT usually occurs in the supratentorial compartment. The extensive collateral network leads to its extremely rare occurrence in the posterior fossa [2]. Veins of cerebellum are reportedly divided into superficial and deep groups. The superficial veins is mainly responsible for draining the cortical surfaces of cerebellum, and the deep veins transits in the fissures between brainstem and cerebellum [14, 15]. The superior cortical surface of the cerebellum is drained by the superior vermian veins and the superior hemispheric veins. These veins empty into the great vein of Galen in the midline and medial and lateral transtentorial sinuses, finally combining with the transverse and straight sinus. The suboccipital surface and the posterior inferior cortical surface of cerebellum are drained by the inferior hemispheric veins and inferior vermian veins. These hemispheric veins also empty into the transverse sinus via the transtentorial sinus [16]. Due to the complicated anatomical variability of posterior fossa veins and the limitation of MRV imaging, it is quite difficult to access the exact location of criminal vascular area and distinguish hypoplastic sinus from thrombosis by using the TOF MRV technique alone. Digital subtraction angiography (DSA) shows unique advantages in displaying the venous malformation, delayed drainage and flow direction. Moreover, the contrast-enhanced 3D T1-MPRAGE sequence is not influenced by the angle between vessel and scan slab or flow velocity, therefore it can provide excellent delineation of venous structures and good contrast resolution between normal sinuses and adjacent lesions [17]. In the current report, contrast-enhanced 3D T1-MPRAGE revealed both the superior and inferior hemispheric veins of left cerebellum in the patient. Furthermore, DSA showed that the left transtentorial sinus was not visualized, and the cortical veins in the left cerebellar hemisphere were tortuous and dilated, which finally drained through the right transtentorial sinus with delayed drainage. These imaging findings suggested that this patient may suffer from thrombosis of the left transtentorial sinus. Our results also confirmed that 3D T1-MPRAGE can be used as an alternative noninvasive technique for the diagnosis and short-term follow-up of patients with sinus thrombosis.

Given the cerebral venous sinus thrombosis, anticoagulation was conducted as the first-line therapy by using either body-weight-adjusted subcutaneous low-molecular weight heparin or dose-adjusted intravenous heparin, with an at least doubled activated partial thromboplastin time. Concomitant intracerebral hemorrhage related to CVT is not considered as a contraindication to heparin therapy [18]. Consistently, our patient had a good prognosis after treatment, which also suggest the diagnosis of CVT.

Possibile diagnosis of isolated CVT should be considered when swelling and enhancing cerebellar lesions in the cerebellum occur. PV maybe a predisposing factors for CVT and the JAK2 V617F mutation is helpful for diagnosis. Contrast-enhanced 3D T1-MPRAGE is able to provide valuable diagnostic information comparable to DSA.

## Data Availability

Data sharing is not applicable to this article as no datasets were generated or analyzed during the current study.

## Abbreviations

ADC: Apparent diffusion coefficient
CVT: Cerebral venous thrombosis
CT: Computerized tomography
DSA: Digital Subtraction Angiography
DWI: Diffusion weighted imaging
FLAIR: Fluid-attenuated inversion recovery
JAK2: Janus Kinase 2
MRI: Magnetic resonance imaging
MRV: Magnetic resonance venography
PV: Polycythemia vera
3D T1-MPRAGE: Three-dimensional T1 magnetization-prepared rapid acquisition gradient echo
TOF: Time-of-flight
T1WI: T1-weighted images
T2WI: T2-weighted images
